# Evolutionarily emerged G tracts between the polypyrimidine tract and 3′ AG are splicing silencers enriched in genes involved in cancer

**DOI:** 10.1186/1471-2164-15-1143

**Published:** 2014-12-19

**Authors:** Muhammad Sohail, Wenguang Cao, Niaz Mahmood, Mike Myschyshyn, Say Pham Hong, Jiuyong Xie

**Affiliations:** Department of Physiology, University of Manitoba, 440 BMSB, 745 Bannatyne Avenue, Winnipeg, MB R3E 0J9 Canada; Department of Biochemistry & Medical Genetics, Faculty of Medicine, University of Manitoba, Winnipeg, MB R3E 0J9 Canada

**Keywords:** 3′ splice site, Alternative splicing, G-tract, Evolution, Cancer

## Abstract

**Background:**

The 3′ splice site (SS) at the end of pre-mRNA introns has a consensus sequence (Y)_n_NYAG for constitutive splicing of mammalian genes. Deviation from this consensus could change or interrupt the usage of the splice site leading to alternative or aberrant splicing, which could affect normal cell function or even the development of diseases. We have shown that the position “N” can be replaced by a CA-rich RNA element called CaRRE1 to regulate the alternative splicing of a group of genes.

**Results:**

Taking it a step further, we searched the human genome for purine-rich elements between the -3 and -10 positions of the 3′ splice sites of annotated introns. This identified several thousand such 3′SS; more than a thousand of them contain at least one copy of G tract. These sites deviate significantly from the consensus of constitutive splice sites and are highly associated with alterative splicing events, particularly alternative 3′ splice and intron retention. We show by mutagenesis analysis and RNA interference that the G tracts are splicing silencers and a group of the associated exons are controlled by the G tract binding proteins hnRNP H/F. Species comparison of a group of the 3′SS among vertebrates suggests that most (~87%) of the G tracts emerged in ancestors of mammals during evolution. Moreover, the host genes are most significantly associated with cancer.

**Conclusion:**

We call these elements together with CaRRE1 *regulatory RNA elements between the Py and 3′AG* (REPA). The emergence of REPA in this highly constrained region indicates that this location has been remarkably permissive for the emergence of *de novo* regulatory RNA elements, even purine-rich motifs, in a large group of mammalian genes during evolution. This evolutionary change controls alternative splicing, likely to diversify proteomes for particular cellular functions.

**Electronic supplementary material:**

The online version of this article (doi:10.1186/1471-2164-15-1143) contains supplementary material, which is available to authorized users.

## Background

Alternative splicing is the major source of proteomic diversity in vertebrates [1–3]. It occurs in more than 90% of human genes and plays important roles in cell function and the development of diseases [4–8]. The usage of alternative exons is tightly regulated in cells in a spatial and temporal manner by *cis*-acting RNA elements and *trans*-acting factors [9, 10]. *Cis*-acting elements have been identified around or overlapping with motifs of the splice sites. It is however very rare for a *de novo* regulatory element to exist between the polypyrimidine tract and 3′AG of the 3′ splice site (3′SS), apparently due to the constrained sequence and space of the motifs for proper splicing [11–13].

The 3′ splice sites at the end of introns have a highly conserved arrangement of consensus sequence (Y)_n_NYAG [13], where the polypyrimidine tract (Py) is close to the 3′ AG for the binding of the heterodimmeric U2AF65 and U2AF35 [13], respectively. The space between the (Y)n and 3′ AG is highly constrained: simply increasing the distance weakens splice site usage [14].

We have shown previously that a CA-rich element called CaRRE1 is within this 3′SS space and bound by hnRNP L to regulate depolarization-induced splicing [15–20], but it is not clear whether a purine-rich element could be tolerated at this location. One group of the well-characterized purine-rich splicing regulatory elements are guanidine (G) tracts containing a minimal functional G_3_ [21], mostly as silencers in exons or enhancers in introns [21–23]. Here we searched the human genome for such potential elements and report the identification and characterization of a large group of G tracts between the (Y)n and 3′ AG, which we call with other elements at this special location REPA (*regulatory RNA elements between the Py and 3′AG*).

## Results

### REPA G tracts within a group of human 3′SS

**Figure 1:**
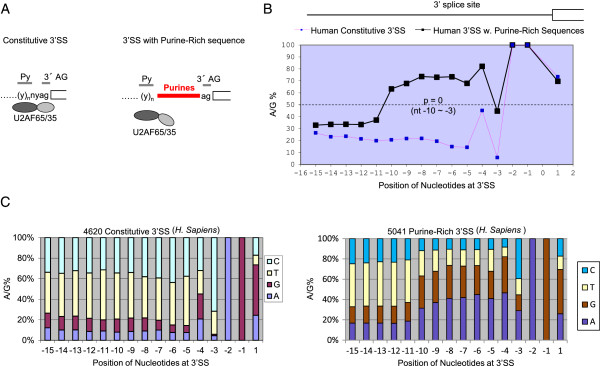
**Identification of a large group of 3′SS that contain Purine-rich sequences between the -3 and -10 positions in front of the 3′AG. A**. Diagram showing the location of purine-rich sequences (Pu) between the polypyrimidine tract (Py) and 3′ AG of constitutive exons in the human genome. The U2AF65/35 heterodimer, which normally bind the Py and 3′ AG, respectively, could be disrupted by the presence of Purine sequences between them. **B**. Percent of A/G at the 3′SS of human constitutive 3′SS (dotted pink line, n = 4620 3′SS) or 3′SS with Pu-rich elements (black solid line, n = 5041 3′SS) as in **A**. The p values, obtained by hypergeometric test, are 0 for all the nucleotide positions between -10 and -3. **C**. Percent of A/G shown in the context of individual nucleotides at each position of the 4620 constitutive (Left) or Pu-rich element-harboring (Right) 3′SS.

To determine if there are purine-rich REPA like the CaRRE1, we searched the 3′SS of annotated introns in the human genome for those with >60% A/G content within the -10 ~ -3 nucleotides of the intron end (Figure 1A-B), a length sufficient to harbor a splicing regulatory RNA element. This identified 5,041 unique 3′SS of 3′AG introns. On average, the A/G content between the -10 and -5 positions is about 70% compared to only about 20% for that of constitutive exons (Figure 1B-C). At the -4 and -3 positions, the A/G content is also higher, about 40% over the constitutive ones. Overall, the A/G content between the -10 and -3 positions is significantly higher (p = 0 for each position in hypergeometric test) than that of constitutive exons.

**Figure 2:**
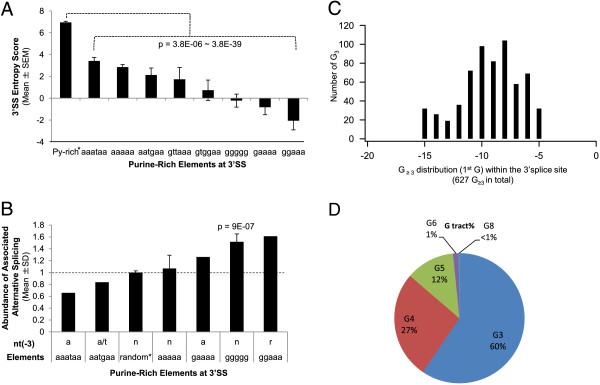
**The sequences of the purine-rich-element-harboring 3′SSs deviate from the constitutive 3′SS (A) and a group of them are significantly associated with alternative splicing (B-D). A**. Entropy scores of the 3′SS. n = 3847, 76, 411, 71, 35, 46, 125, 131, 99 of 3′SS for columns from left to right, respectively. *: of constitutive exons from the human genome, which are overall Py-rich (Figure 1B). The range of the Student’s t-test p values of the constitutive versus each of the other groups of splice sites containing purine-rich elements are indicated. **B**. Abundance of alternative splicing associated with different 3′SS sequence between the Py and 3′AG. For this graph, the percentages of alternative exons relative to the total number of exons in each group were first obtained by analysis in the UCSC Genome Browser, ranging from 33% for the aaataa to 82% for the ggaaa groups, respectively. The percentage for the group of randomly chosen exons was taken as the baseline abundance 1 (dotted line); the percentages for the other groups were all normalized to it. *: a group of exons randomly chosen from the human genome. nt(-3): nucleotide at the -3 position of 3′splice sites, which is a “y” for constitutive sites. n: any nucleotide, r: purines A or G. n = 24, 63, 169, 162, 36, 121, 38 of 3′SS for columns from left to right, respectively. The p value was obtained by hypergeometric test for the abundance of alternative splicing of the G pentamer group in the whole population of 3′ splice sites examined. **C**. Histogram showing the distribution of the first Gs of G tracts of 627 alternative exons within the 3′SS. The first Gs peak at -10 and -8. **D**. Pie-shaped distribution of G tracts of the 737 alternative exons ranging from 3 to 8 Gs in a run, with G_3–5_ comprising ~98%.

To identify consensus elements, we examined the nucleotides -15 to -3 nt to allow for a maximal 5 upstream nucleotides to form elements with the search target sequence. These sequences were subject to the MEME (multiple Em for motif elicitation) analysis as reported previously [24, 25]. This identified G-rich motifs, among others (Figure 2), as one of the top consensus elements.

### The REPA G-tracts are highly associated with alternative splicing

To determine the strength of the splice sites containing the purine-rich elements, we calculated their MaxEntScan entropy scores, as described by Yeo and Burge [26]. The scores are significantly lower than the 3847 3′SS randomly chosen from the human genome, with those containing the G pentamer and GGAAA among the lowest (Figure 2A, p = 3.8E-06 ~ 3.8E-39). Therefore, the purine-rich REPA likely weakens the 3′SS.

To determine whether these 3′SS are associated with alternative splicing, we examined some of the REPA-containing 3′SS and their downstream exons of human mRNA or expressed sequence tags (EST) in the UCSC Genome Browser. The abundance of associated alternative splicing showed a general trend consistent with entropy scores, for example, higher abundance among exons with 3′SS containing the G pentamer (Figure 2B, p = 9E-07). However, further comparison among three groups of 3′SS (Figure 1A, gtggaa, ggggg and gaaaa groups) with similarly low entropy scores (around 0, p > 0.05 by t-test) indicated that not all of them were associated with higher abundance of alternative splicing. For instance, the gaaaa group had only about half of the G pentamer group in the abundance. Thus, specific purine sequences are likely also associated with the high abundance of alternative splicing.

We then chose to focus on the REPA G tracts. We examined in total 922 REPA G_3–8_-harboring 3′SS and found 627 of them (68%) associated with alternative splicing events (Additional file 1: Table S1). The first G mainly starts between the -11 and -6 positions (Figure 2C), and about 99% of them are G_3–5_(Figure 2D).

**Figure 3:**
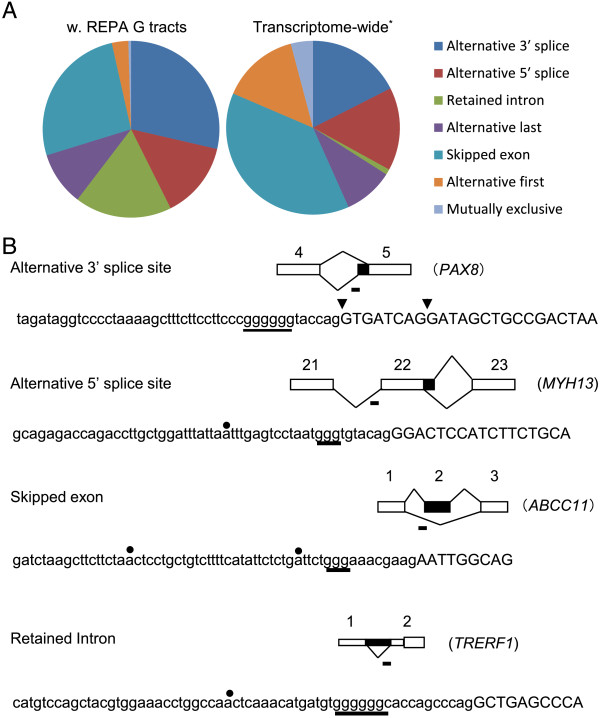
**The REPA G tracts-associated types of alternative splicing and examples of exons (genes) with the major types. A**. Pie-shaped distribution of the relative abundance of different types of alternative splicing associated with the 627 REPA G tracts. The transcriptome-wide data was based on that by Wang et al., *Nature*’08. The biggest increase in the G tract group is the retained intron over that in the human transcriptomes. **B**. Examples of the major types of alternative splicing associated with the 3′SS G tracts. The exon numbers or gene names are indicated above or to the right of the splicing diagrams. Horizontal bar: G tract locations. Black boxes: alternative exons/introns. Narrower boxes: untranslated regions of mRNA. The intron/exon boundaries of alternative 3′ splice sites are indicated by arrowheads. The G tracts or G/A-rich motifs are underlined in the sequences. Black dots: potential branch points within the consensus “curay”.

Major types of the associated alternative splicing events include alternative 3′SS or 5′SS usage, cassette exon and intron retention (Figure 3A). Of these, intron retention has the most enrichment, about 17 times of that surveyed in the human transcriptomes of different tissues [5]. The other enriched type is alternative 3′ splice (1.6 times). Examples of these types of alternative splicing and their G tracts are shown in Figure 3B: the alternative 3′ splicing of the exon 5 of *PAX8* (*paired box 8*), alternative 5′ splicing of the exon 22 of *MYH13* (*myosin, heavy chain 13, skeletal muscle*), skipped exon (cassette) 2 of *ABCC11* (*ATP-binding cassette, sub-family C (CFTR/MRP), member 11*) and retained intron 1 of *TRERF1* (*transcriptional regulating factor 1*).

### The REPA G-tracts are splicing silencers

**Figure 4:**
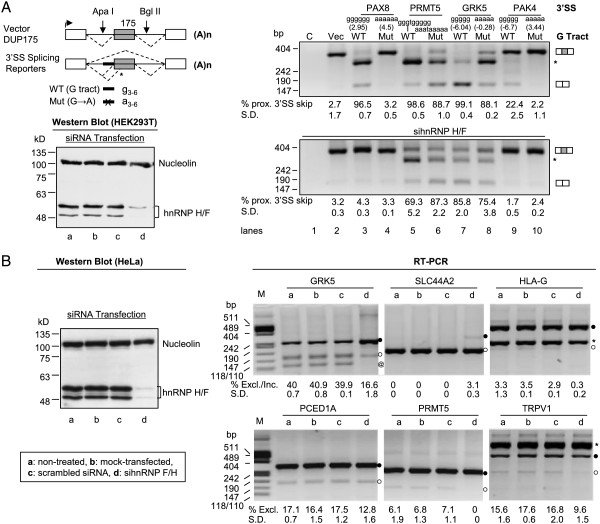
**The REPA G tracts are splicing silencers and their related exons are controlled by G tract-binding splicing factors hnRNP H/F in human cells. A**. (Upper left) Diagram of splicing reporter mini-genes and (Lower left) western blot of RNA interference samples of HEK293T cells using anti-nucleolin and anti-hnRNP H/F antibodies. (Right) agarose gels of RT-PCR products from transiently expressed reporters containing the G-tract-harboring 3′SS or its G- > A mutants in normal (Above) or RNA interference (Below) human HEK293T cells. Boxes: exons, horizontal lines: introns. Splicing pathways are indicated by dashed lines above or below the pre-mRNA. Heavy black bar: 3′SS with REPA G tract (WT) or its G- > A mutant (Mut). ApaI and BglII: restriction-cloning sites. *: cryptic splicing pathway through the distal 3′SS or its product. The percentages (mean ± S.D., n = 3) of molar amounts of the proximal (prox., upstream the cryptic site) 3′SS- skipped product are indicated below each lane. In the brackets above the lanes are the MaxEnt scores of the G tract or mutant 3′ splice sites of the minigenes. For PRMT5, the scores are -6.01 and -2.39, respectively. The vector DUP175 has a score of 9.50. **B**. (Left) western blot of RNA interference samples of HeLa cells as in **A**. (Right) Agarose gels of RT-PCR products of endogenous REPA G-tract-harboring genes in HeLa cells. The percentages (%, mean ± S.D., n = 3) of molar amounts of the minor isoforms (exon excluded “Excl.” except SLC44A2 as inclusion “Incl.”) are below each lane. Black dots: exon included, open circle: exon excluded. @: a shorter product consistent with a variant in frog. *: non-specific products by sequencing results.

To determine the role of the G-tracts in alternative splicing, we examined four REPA G tract-harboring 3′SS by transferring them to the upstream of a constitutive exon derived from the human beta-globin gene (Figure 4A, upper left). Three of them almost abolished the usage of the immediate downstream (proximal) 3′AG and activated substantial usage of a further downstream cryptic 3′SS within the exon or caused exon skipping (upper right, lanes 3, 5 and 7). The other one reduced the proximal 3′AG usage by about 22.4% (lane 9). Importantly, mutating the Gs to As of the REPA G tracts almost completely (lanes 4 and 10) or partially (lanes 6 and 8) restored the usage of the proximal 3′AG, accompanied by abolished (lane 4), reduced (lane 6) or increased (lane 8) cryptic splicing. This suggests that the G tracts are more than just a purine-rich spacer between the Py and 3′AG, which also weakens the 3′ splice site (see the MAXENT score and also Figure 2A). The G tracts inhibit the usage of proximal 3′AG but often activate distal 3′AG, consistent with their increased association with alternative 3′SS (Figure 3A) and the dependence of the effect on their location relative to the target splice site. Moreover, the ΔGs of the potential secondary structures caused by the mutations, as tested by Mfold [27], do not correlate with the splicing changes, suggesting that the mutation effect is not due to a secondary structural change. Therefore, we conclude that all four REPA G tracts tested are splicing silencers with the Gs playing critical roles.

In order to examine the role of known G tract-binding factors hnRNP H/F in splicing through the REPA G tracts, we carried out RNA interference assays (Figure 4A, lower panel). Two of the reporters PAX8 and PAK4 almost completely lost the G tract silencer effect upon knockdown of hnRNP H/F (lanes 3 & 9) whereas the other two exhibited about 29.3% and 13.3% decrease in exon skipping (lanes 5 & 7). This is accompanied by abolished (lane 3), reduced (lane 5) or increased (lane 7) level of the cryptic splice product. Notably, the mutated mini-genes showed almost no change in exon skipping (lanes 4, 6 & 10) except one that showed about 12.7% decrease (lane 8) but still 10.4% less than its wild type. The mutants also showed reduced cryptic splicing in the cases of PRMT5 and GRK5 (lanes 6 and 8), suggesting that usage of the cryptic splice sites are also affected by other hnRNP H/F target element(s) in the reporter. Hence, the silencer effect of REPA G tract on exon skipping or its activation of cryptic 3′SS usage is mainly through hnRNP H/F.

To further validate the G tract silencer effect on splicing of endogenous exons, we used RNA interference of hnRNP H/F in human HeLa cells and measured the effect of loss-of-function of these factors on exon usage (Figure 4B). We tested 8 exons of the above 4 and 4 additional genes. We were able to successfully detect expression and alternative splicing of 6 exons in this cell line (Figure 4B). All of them showed an increased exon inclusion (SLC44A2) or a reduced exon skipping (of the minor isoform) upon hnRNP H/F knockdown (p < 0.001). Thus, the G-tract-binding factors hnRNP H/F are essential repressors of all of the six exons of endogenous genes tested, consistent with the silencing effect of the G tracts in splicing reporters (Figure 4A).

### The REPA G tracts emerged mostly in the ancestors of mammals during evolution

To determine the evolutionary origin of the G tracts, we analyzed 22 3′SS in multiple species ranging from fish to humans. We were able to determine the presence or absence of 15 of them in every class of animals analyzed (Table 1). G tracts of 10 of these 3′SS (~67%) were also found early as in marsupials, four of them (~27%) in other mammals and only two of them in birds, reptiles and fish. Therefore, it is likely that most of the G tracts (~87%) emerged in ancestors of mammals.

**Figure 5:**
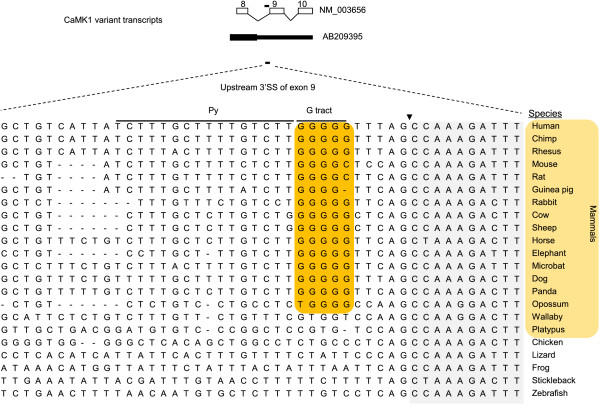
**An example of evolutionary emergence of REPA G tracts between the polypyrimidine tract and 3′AG within the upstream 3′SS of alternative exons of mammalian genes.** Shown is an alignment of sequences around the 3′SS of the *CaMK1* gene from 22 species. The G tract (black bar in diagram) is at the 3′SS of intron 8 (in front of the exon 9). Retention of both upstream and downstream introns around exon 9 generates a splice variant with an alternative stop codon in the upstream intron (Genbank accession number **AB209395**). In most other transcripts, both introns are spliced. Arrowhead: intron/exon boundary. It can be seen from the alignment that the G pentamer is present in most mammals. It is absent in wallaby, platypus, birds and the other vertebrates shown here.

As an example, the mammal-specific emergence of the G pentamer in CaMK1 is shown in Figure 5. Besides the full length CaMK1, there is a shorter transcript (GenBank accession # AB209395) with the retention of introns 8 and 9 within the 3′ untranslated region (UTR, Figure 5). The G pentamer GGGGG is at the upstream 3′SS of exon 9, where it might weaken the 3′SS to promote intron retention.

**Table 1:** **The REPA G tracts among vertebrates**

#	Gene symbol	Transcript ID	Exon rank	Exon size (bp)	3′SS -15 ~ -1 nt	Presence (1) or Absence (0) of REPA G tract				
						Other mammals	Marsupials	Birds	Reptiles	Fish
1	CAMK1	ENST00000256460	9	79	gtcttgggggtttag	1	1	0	0	0
2	CNOT3	ENST00000358389	3	276	gttctgtgggggcag	1	1	0	0	0
3	DUSP12	ENST00000367943	5	187	gtttgggggttgcag	1	1	0	0	0
4	FES	ENST00000328850	6	138	ccgtctcgggggcag	1	1	0	0	0
5	TRPV1	ENST00000174621	7	180	gctccccgggggcag	1	0	0	0	0
6	SLC44A2	ENST00000380614	22	104	ccggggggagcccag	1	1	0	0	0
7	RQCD1	ENST00000273064	7	92	ttttgggggaaacag	1	1	0	0	0
8	PFN2	ENST00000239940	2	193	tttggtggggggcag	1	0	0	0	0
9	PDZK1	ENST00000339729	6	197	gttgggggagggtag	1	1	0	0	0
10	LRRC23	ENST00000007969	6	137	gccctgggggtctag	1	0	0	0	0
11	LAMB1	ENST00000222399	22	225	tttatcgggtgacag	1	0	0	0	0
12	C3orf62	ENST00000343010	2	92	acctgggggctgcag	1	1	0	0	0
13	PRMT5	ENST00000324366	3	86	ggtgggggagtgcag	1	1	0	0	0
14	PAOX	ENST00000368534	2	159	tgtttgcgggggaag	1	1	1	1	1
15	METTL13	ENST00000367736	2	176	cctgggcgggggcag	1	1	1	1	
16	MICA	ENST00000364810	4	288	gctgggtgggggcag	1	1	0	ND	ND
17	FAM113	ENST00000309144	4	239	ggggggggtgggtag	1	1	ND	0	0
18	PAX8	ENST00000263334	5	176	cccggggggtaccag	1	1	ND	0	0
19	ABCC11	ENST00000356608	2	117	ttctgggaaacgaag	1	0	ND	0	0
20	HLA-G	ENST00000383504	3	276	aaggggtggggccag	1	1	ND	ND	ND
21	HLA-H	ENST00000360432	3	276	cgggggcggggccag	1	1	ND	ND	ND

ND: not determined.

Alignment of the CaMK1 3′SS from 22 species indicates that the G pentamer (or tetramer in two species) is present in mammals from human to opossum but not in wallaby and platypus, nor is it present in chicken, lizard, frog or fish. Interestingly, there are three Gs in wallaby and two Gs in platypus, both interrupted by a single T. It thus seems that the G tracts emerged in mammalian ancestors but were lost in some marsupials.

The mostly mammalian emergence of the REPA G tracts is consistent with the conclusion that G triplets are more abundant in mammalian than in fish introns as previously reported in a genome-wide study [28].

Taken together, the examined REPA G tracts are mostly mammalian gene-specific, suggesting that they may have evolved to inhibit the splicing of a group of mammalian exons.

### The REPA G tract-host genes are significantly associated with cancer

**Figure 6:**
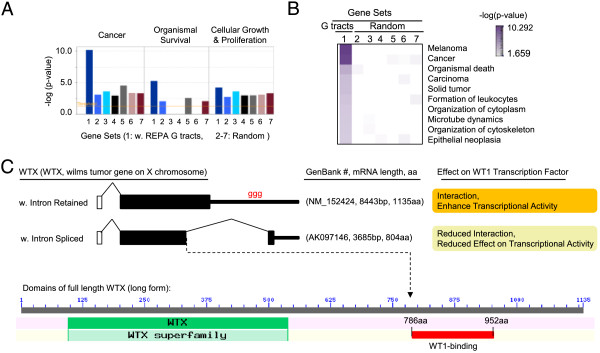
**Significantly clustered functions of the REPA G tract-harboring genes. A**. Shown is a bar graph of the –log (p-value) for the significance level of the functional comparison of 578 genes of the 627 3′SS (left most dark blue column in each functional category) with six random sets (2^nd^ -7^th^ column in each category) of 1319 human genes each. Brownish horizontal line: significance threshold of the functions/diseases of these genes above the background levels (random events) in the Ingenuity database. **B**. Heat map of the different cancers/biological functions with top log p values of enrichment. A scale bar with the color intensity/-log p value is to the upper right corner of the map. **C**. The WTX gene’s REPA G tract and alternative splicing as an example of genes involved in cancer whose alternative splicing (intron retention) causes a functional impact on gene function. The intron retention or splicing results in two isoforms of different lengths with different interaction with WT1 and effects on the latter’s transcriptional activity (Upper panel). The shorter isoform lacks the WT1-interaction domain at the COOH terminus (Lower panel).

To determine whether these G tract-harboring 3′SS are associated with any specific biological functions or diseases, we analyzed 578 mapped genes of the 627 3′SS in the dataset in the Ingenuity Pathway Analyses. In this search, we also included 6 sets of randomly chosen human genes from the ENSEMBL genome database to control the specificity of the analysis. The result indicates that the 578 genes are markedly enriched for genes involved in cancer (444 genes in total, Figure 6, and see also Additional file 1: Tables S2-S3 for a complete list), by about 4 fold -log(p-value) over the 6 sets of randomly chosen genes. Particularly melanoma, carcinoma and solid tumors have the most significant enrichment of these genes, including *ABCC11 (melanoma drug resistance)*, *SNRNP200 (retinitis pigmentosa)*, *TRERF1* (breast cancer) and WTX (*Wilms tumor gene on X chromosome, Wilms tumor*) [29–33]. SNRNP200 together with another seven genes encode splicing factors: *HNRNPH1, HNRNPH3, KHSRP, MBNL1, PPAN, PRPF8, SNRPN*. Particularly interesting is the hnRNP H1 and H3, which are G tract-binding proteins themselves, predicting an auto-regulatory circuit through alternative splicing. Moreover, most of the genes in Figures 3, 4 and 5 (except PCED1A) are involved in cancer as well (see also Additional file 1: Table S3). Furthermore, similar Ingenuity pathway analysis using the full set of the G_3–8_ 3′SS-containing genes reached the same conclusion. Therefore, the REPA G tracts are significantly enriched in genes involved in cancer, likely to control their alternative splicing and cancer properties.

As an example of the genes involved in cancer, the *WTX* (or called *AMER1, APC membrane recruitment protein 1*) has a retained intron with a 3′SS G_3_ in its second exon [34] (Figure 6C). The full length transcript encodes an 1135aa protein that interacts with the transcription factor *WT1* (*Wilms tumor 1*) and efficiently enhances its transcriptional activity [30, 35]. The intron splicing results in a shorter protein of 804aa lacking the WT1-interacting domain. The shorter isoform has reduced effect on WT1 transcriptional activity [30, 35]. Therefore, the 3′ REPA G tract-associated intron retention/splicing determine the high or low activity of the tumor suppressor WTX. Presence of the G tract appears to favor the full activity of the protein, likely by weakening the 3′ splice site and thus promoting intron retention.

Also enriched are biological functions associated with organismal survival (about 2.5 fold –log(p-value)), and to some extent cellular growth & proliferation (Figure 6A-B, and Additional file 1: Table S2).

Taken together, more than six hundred 3′SS that harbour REPA G tracts associated with alternative splicing are identified in the human genome. Instead of being enhancers as the other mammalian intronic G tracts, these specially localized G tracts appear to be intronic splicing silencers and mostly emerged in the ancestors of mammals. Moreover, their host genes are highly associated with cancer. These data suggest that mammals have evolved *de novo* RNA elements within this highly constrained space of 3′ splice site to alter the mRNA processing step for diverse proteins in particular cellular functions.

## Discussion

### Regulatory RNA elements between Py and 3′AG (REPA)

The splicing regulatory elements can be outside or overlap with the splice site motifs. However, since the location and sequences of the polypyrimidine tract and 3′ AG have been more or less constrained for many splice sites, emergence of *de novo* regulatory elements between them has not been observed frequently. A G-rich sequence was identified in a group of drosophila genes but it was enriched further upstream (60nt) in the intron to inhibit splicing [24]. While the previously reported CaRRE1 of the STREX exon support the existence of REPA elements [16], here the identification of a large group of 3′SS G tracts clearly demonstrate that the emergence of *de novo* regulatory elements do occur at this location, apparently for the regulation of alternative splicing mainly in mammals. Moreover, these elements are location-dependent silencers of the immediate downstream 3′AG (Figure 4A), instead of enhancers as found for the other mammalian intronic G tracts outside of the region between Py and 3′AG [21, 22, 24, 36]. Beyond G tracts, there are also other purine-rich elements identified in this search that await further detailed analysis to reveal their function in splicing regulation. Furthermore, this also implies the existence of other non-purine-rich elements. In fact, we have also observed UGCAUG elements at this location of alternative exons (Xie, unpublished observation). Therefore, the repertoires of REPA will apparently increase in the future. Overall, the REPA provides a distinct example for the variations of splice sites to cause alternative usage of exons [37].

### Variations of splice sites for alternative splicing and proteomic diversity during evolution

Splicing regulatory RNA elements have evolved from fish to humans with dramatic emergence of some additional elements including G tracts [28]. Here the REPA G tracts appear to have evolved mostly in mammalian ancestors, evident from the alignment of 3′SS of multiple species (Figure 5 and Table 1). The evolved elements likely increase the possibility for alternative splicing and cause functional changes of the proteins (Figure 6). Moreover, their recruitment of trans-acting factors, such as hnRNP H/F (Figure 4), would make it possible for upstream signaling to reach the target splice site [38], adding a further layer to the splicing regulation. With the help of more REPAs in the genome (Figures 1 and 2), more diverse protein isoforms and functions of mammalian genes could be generated.

### Role of G tracts and alternative splicing in cancer

Accumulating evidence indicate that alternative splicing has been exploited by cancer cells to favor their survival [39, 40]. G tracts have been found to control the alternative splicing of genes involved in cancer, such as Bcl-x [22, 41]. Their *trans*-acting factors have also been found to regulate alternative splicing during tumorigenesis. For instance, upregulation of hnRNP H in gliomas drives the IG20 to anti-apoptotic MADD (MAP-kinase activating death domain protein) splicing in favor of invasiveness [42]. In HeLa cells, hnRNP H/F upregulates the pro-apoptosis variant Bcl-xS [41]. Multiple G-tracts can also form a stable four strand structure called a G quadruple that is less represented in exon sequences [43]. They are formed in a cell-cycle-dependent way with the highest abundance in the S phase [44], and have been proposed as therapeutic targets for diseases including cancer, for example, by controlling oncogene transcription [45, 46]. They are also proposed to control alternative splicing of the tumor suppressor p53 [47], and the telomere RNA hTERT [48]. Multiple copies of G tracts are also seen in some of the 3′SS examined here. For example the 3′SS of exon 4 of the matrix-remodeling associated 8 (*MXRA8*) gene has five copies of G_2–5_ within 50 nt of the intron end. Whether G quadruples may form and play a role in the control of alternative splicing of some of these cancer-related genes will need future studies.

In summary, the identification of hundreds of human 3′SS containing the REPA G tracts and the confirmed splicing silencer effect of representative ones suggest that the highly constrained 3′SS region has been permissive for the emergence of *de novo* regulatory elements for splicing control of a large group of mammalian genes. Their significant enrichment in genes involved in cancer implies that their regulation of splicing perhaps control certain properties of cancer cells.

## Conclusions

A large group of human 3′SS have evolved purine-rich elements between the Py and 3′AG. Particularly the G tract elements are splicing silencers requiring hnRNP H/F for their activity. Such G tract-harboring human genes are significantly enriched for functions related to cancer. These data demonstrate that the often highly constrained space between the Py and 3′AG has been remarkably permissive for the evolution of splicing regulatory elements, likely to diversify protein functions in mammals. The presence of such elements at the 3′SS is also predictive of alternative splice sites.

## Methods

### Plasmid construction

Splicing reporters with 3′SS REPA or human G-tract mutant were created by replacing the corresponding upstream 3′SS of the middle constitutive exon of DUP175 as carried out previously [49].

### Cell culture and transfection

HEK293T cells were grown in Dulbecco’s modified Eagle’s medium containing 10% new-born calf serum and 1% penicillin/streptomycin/glutamine solution (Invitrogen). Transfection of overnight cultures of HEK293T cells was performed with Lipofectamine 2000 (Invitrogen) according to manufacturer’s protocol, using 0.25 μg of splicing reporter minigene plasmids in 12-well plates. Transfection of siRNA into HeLa cells was done with Lipofectamine RNAiMAX (Invitrogen) according to manufacturer’s instructions.

### RNA interference

To knock down hnRNP H/F in HeLa cells, we synthesized siRNA targeting the 20 nucleotide sequence GCGACCGAGAACGACAUUU [41]. HeLa cells were transfected with 360 pmoles of siRNA twice in 6-well plates with 24-hour intervals. Cells were harvested after 72 hours of first transfection for protein and RNA analysis.

### RT-PCR

We performed semi-quantitative RT-PCR of endogenous genes based on previously described procedure [15]. Specifically, in 10 ul reverse transcription reactions we used 250 ng of cytoplasmic RNA and used 0.75 ul of it in a 12.5 ul PCR reaction for 30 cycles (24 for mini-genes) with annealing temperature of 60°C. We quantified bands intensities using ImageJ (National Institutes of Health).

### Human genome search

We wrote a bioperl script (Additional file 2: REPA_Perl) to search the 3′SS in the annotated EMSEMBL human genome for purine rich motifs within -10 ~ -3 position at intron ends. The consensus elements were identified using the -15 ~ -3 nucleotides by the MEME program as described previously [25]. The MaxEntScan entropy scores of 3′SS were determined as described [26], using 23 nt (20 nt intron and 3 nt exon) of the 3′SS at the Burge lab website (http://genes.mit.edu/burgelab/maxent/Xmaxentscan_scoreseq_acc.html). Human genome sequence was downloaded from the ENSEMBL website (http://uswest.ensembl.org/index.html). HGNC symbols of genes containing identified 3′ spice sites were retrieved from Biomart (http://www.biomart.org/index.html) using ENSEMBL gene IDs. From these 3′SS, we determined the alternative exons by alignment with that in the UCSC Genome Browser (http://www.genome.ucsc.edu/index.html); those without evidence for the presence of splice sites were excluded from further analysis. We used ENSEMBL gene IDs to obtain the clusters of identified genes involved in same functions/diseases using Ingenuity Pathway Analysis application (http://www.ingenuity.com).

### Statistical analyses

Entropy scores were compared with two-tailed Student’s t-test in Figure 2A. Ingenuity pathway analysis was by right-tailed Fisher’s exact test in Figure 6B. The abundance of purine nucleotides or alternative splicing was compared by hypergeometric test in Figures 1B and 2B, respectively.

### Notes

No ethics approval was required for the research conducted for this manuscript.

## Electronic supplementary material

Additional file 1: **Tables S1, S2 and S3.** (XLSX 354 KB)

Additional file 2: **REPA_Perl.** (PDF 11 KB)
