# Supplementary material for: Evolutionarily emerged G tracts between the polypyrimidine tract and 3′ AG are splicing silencers enriched in genes involved in cancer
Source: BMC Genomics. 2014 Dec 19;15(1):1143. doi: 10.1186/1471-2164-15-1143 (PMC4320613; doi:10.1186/1471-2164-15-1143)
Supplement: Supplementary file 2 — Additional file 2: REPA_Perl. (PDF 11 KB) [file 12864_2014_6922_MOESM2_ESM.pdf]

```
use Bio::Seq;
use Bio::SeqIO;

$output=shift;

my $in = Bio::SeqIO->new(-file => "human_int_ex.txt",
-format=>'genbank');
open(OUT,">$output.txt")||die"could not open output file $!\n";
#my $out = Bio::SeqIO->new(-file => ">homo.fa", -format => 'fasta');

while(my $seq = $in->next_seq){
#my $seq = $in->next_seq;
my $intron_exon;
my $gene;
my $note;
for my $feature($seq->get_SeqFeatures){

  if ($feature->location->isa('Bio::Location::SplitLocationI')
                && $feature->primary_tag eq 'mRNA'){

    if($feature->has_tag('gene')){
      for my $value($feature->get_tag_values('gene')){
        $gene=$value;
      }
    }
#    print $gene, "\n";
    if($feature->has_tag('note')){
      for my $name($feature->get_tag_values('note')){
        $note=$name;
      }
    }
#    print $note, "\n";

    my @start=();
    my @end=();
    my @exonlength=();
    my $strand;
    my $count=0;
    foreach my $location ($feature->location->sub_Location) {
        $start[$count]=$location->start;
        $end[$count]=$location->end;
        $exonlength[$count]=$end[$count]-$start[$count]+1;
        $strand=$location->strand;
        $count++;
        }

        my @length=();
        for ($j=0;$j<$count-1;$j++){
          if ($strand < 0){
            my $len=($start[$j]-1)-($end[$j+1]+1)+1;
            $length[$j]=$len;
          }else{
```

```
    my $len=($start[$j+1]-1)-($end[$j]+1)+1;
    $length[$j]=$len;
  }
}

my @new_start=();
my @new_end=();
if ($length[0]>500){
  if ($strand < 0){
  $new_start[0]=$start[0]-500;
  $new_end[0]=$end[0];
  }else{
  $new_start[0]=$start[0];
  $new_end[0]=$end[0]+500;
  }
}else{
  if ($strand < 0){
  $new_start[0]=$start[0]-$length[0];
  $new_end[0]=$end[0];
  }else{
  $new_start[0]=$start[0];
  $new_end[0]=$end[0]+$length[0];
  }
}

for ($j=0;$j<$count-1;$j++){
  if ($length[$j]>500 && $length[$j+1]>500){
    if ($strand < 0){
      $new_start[$j+1]=$start[$j+1]-500;
      $new_end[$j+1]=$end[$j+1]+500;
    }else{
    $new_start[$j+1]=$start[$j+1]-500;
    $new_end[$j+1]=$end[$j+1]+500;
    }
  }
  if ($length[$j]>500 && $length[$j+1]<=500){
    if ($strand < 0){
      $new_start[$j+1]=$start[$j+1]-$length[$j+1];
      $new_end[$j+1]=$end[$j+1]+500;
    }else{
    $new_start[$j+1]=$start[$j+1]-500;
    $new_end[$j+1]=$end[$j+1]+$length[$j+1];
    }
  }
  if ($length[$j]<=500 && $length[$j+1]>500){
    if ($strand < 0){
       $new_start[$j+1]=$start[$j+1]-500;
       $new_end[$j+1]=$end[$j+1]+$length[$j];
    }else{
    $new_start[$j+1]=$start[$j+1]-$length[$j];
    $new_end[$j+1]=$end[$j+1]+500;
    }
```

```
            }
            if ($length[$j]<=500 && $length[$j+1]<=500){
              if ($strand < 0){
                $new_start[$j+1]=$start[$j+1]-$length[$j+1];
                $new_end[$j+1]=$end[$j+1]+$length[$j];
              }else{
              $new_start[$j+1]=$start[$j+1]-$length[$j];
              $new_end[$j+1]=$end[$j+1]+$length[$j+1];
              }
            }
          }

          my $id=$seq->display_id();
          my $acc=$seq->accession_number();

          my $downstream;
          my $upstream;
          for ($j=1;$j<$count-1;$j++){
              my $left_intron =
$seq->trunc($new_start[$j],$start[$j]-1);
              my $right_intron=$seq->trunc($end[$j]+1,$new_end[$j]);
              my $exon = $seq->trunc($start[$j],$end[$j]);

              my $Total_AG;
              my $sub_seq;
              if ($strand<0){
                my $l_intron=$left_intron->revcom->seq;
                my $r_intron=$right_intron->revcom->seq;

                $downstream=$left_intron->length();
                if ($downstream<500){
                  $downstream=$downstream;
                }
                else{
                  $downstream=500;
                }
                $upstream=$right_intron->length();
                if ($upstream<500){
                  $upstream=$upstream;
                }
                else{
                  $upstream=500;
                }

                my $e=$exon->revcom->seq;
                $intron_exon=lc($r_intron).uc($e).lc($l_intron);

                if (length($r_intron) >= 10){

$sub_seq=$seq->trunc($end[$j]+3,$end[$j]+10)->revcom->seq;
                }
```

```
            }else{
              my $l_intron=$left_intron->seq;
              my $r_intron=$right_intron->seq;

              $upstream=$left_intron->length();
              if ($upstream<500){
                $upstream=$upstream;
              }
              else{
                $upstream=500;
              }
              $downstream=$right_intron->length();
              if ($downstream<500){
                $downstream=$downstream;
              }
              else{
                $downstream=500;
              }
              my $e=$exon->seq;
              $intron_exon=lc($l_intron).uc($e).lc($r_intron);

              if (length($l_intron) >= 10){
$sub_seq=$seq->trunc($start[$j]-10,$start[$j]-3)->seq;
              }
            }

            @nts=split(//,$sub_seq);
            $a=0;
            $g=0;
            foreach $base(@nts){
              $a++ if $base eq 'A';
              $g++ if $base eq 'G';
            }
            $Total_AG=$a+$g;

            if ($Total_AG > 4){
            print $intron_exon,"\n";

            $i=$j+1;
$seq_id=$id."|".$acc."|".$gene."|".$note."|".$i."|"."exonsize"."|".$
exonlength[$j]."|"."upstream"."|".$upstream."|"."downstream"."|".$do
wnstream."|".$intron_exon;
            print OUT "$seq_id\n";
            }
        }
        if ($strand<0){
            my $right_intron =
$seq->trunc($end[$count-1]+1,$new_end[$count-1])->revcom->seq;
            my $exon =
$seq->trunc($start[$count-1],$end[$count-1])->revcom->seq;
```

```
            my $intron_exon=lc($right_intron).uc($exon);


$upstream=$seq->trunc($end[$count-1]+1,$new_end[$count-1])->length()
;
            if ($upstream<500){
              $upstream=$upstream;
            }
            else{
              $upstream=500;
            }


            if (length($right_intron) >= 10){

$sub_seq=$seq->trunc($end[$count-1]+3,$end[$count-1]+10)->revcom->se
q;
            }
            @nts=split(//,$sub_seq);
            $a=0;
            $g=0;
            foreach $base(@nts){
              $a++ if $base eq 'A';
              $g++ if $base eq 'G';
            }
            $Total_AG=$a+$g;
            if ($Total_AG > 4){
            print $intron_exon,"\n";


$seq_id=$id."|".$acc."|".$gene."|".$note."|".$count."|"."exonsize"."
|".$exonlength[count-1]."|"."upstream"."|".$upstream."|"."downstream
"."|"."0"."|".$intron_exon;
            print OUT "$seq_id\n";
             }
        }else{
            my
$left_intron=$seq->trunc($new_start[$count-1],$start[$count-1]-1)->s
eq;
            my
$exon=$seq->trunc($start[$count-1],$end[$count-1])->seq;
            my $intron_exon=lc($left_intron).uc($exon);


$upstream=$seq->trunc($new_start[$count-1],$start[$count-1]-1)->leng
th();
            if ($upstream<500){
              $upstream=$upstream;
            }
            else{
              $upstream=500;
            }
```

```
            if (length($left_intron) >= 10){

$sub_seq=$seq->trunc($start[$count-1]-10,$start[$count-1]-3)->seq;
            }
            @nts=split(//,$sub_seq);
            $a=0;
            $g=0;
            foreach $base(@nts){
              $a++ if $base eq 'A';
              $g++ if $base eq 'G';
            }
            $Total_AG=$a+$g;
            if ($Total_AG > 4){
            print $intron_exon,"\n";


$seq_id=$id."|".$acc."|".$gene."|".$note."|".$count."|"."exonsize"."
|".$exonlength[count-1]."|"."upstream"."|".$upstream."|"."downstream
"."|"."0"."|".$intron_exon;
            print OUT "$seq_id\n";
            }
       }
   }
  }
}
```
